# Using a Rotating 3D LiDAR on a Mobile Robot for Estimation of Person’s Body Angle and Gender

**DOI:** 10.3390/s20143964

**Published:** 2020-07-16

**Authors:** Dražen Brščić, Rhys Wyn Evans, Matthias Rehm, Takayuki Kanda

**Affiliations:** 1 Graduate School of Informatics, Kyoto University, Kyoto 606-8501, Japan; kanda@i.kyoto-u.ac.jp; 2 Technical Faculty of IT and Design, Aalborg University, 9220 Aalborg, Denmark; revan15@student.aau.dk (R.W.E.); matthias@create.aau.dk (M.R.)

**Keywords:** LiDAR, body angle estimation, gender recognition, social robotics

## Abstract

We studied the use of a rotating multi-layer 3D Light Detection And Ranging (LiDAR) sensor (specifically the Velodyne HDL-32E) mounted on a social robot for the estimation of features of people around the robot. While LiDARs are often used for robot self-localization and people tracking, we were interested in the possibility of using them to estimate the people’s features (states or attributes), which are important in human–robot interaction. In particular, we tested the estimation of the person’s body orientation and their gender. As collecting data in the real world and labeling them is laborious and time consuming, we also looked into other ways for obtaining data for training the estimators: using simulations, or using LiDAR data collected in the lab. We trained convolutional neural network-based estimators and tested their performance on actual LiDAR measurements of people in a public space. The results show that with a rotating 3D LiDAR a usable estimate of the body angle can indeed be achieved (mean absolute error 33.5${}^{{}^{\circ}}$), and that using simulated data for training the estimators is effective. For estimating gender, the results are satisfactory (accuracy above 80%) when the person is close enough; however, simulated data do not work well and training needs to be done on actual people measurements.

## 1. Introduction

What information does a mobile social robot need in a public spaces? In order to move, it needs to know about itself and the space, such as what the space looks like and what its position is inside the space. For interacting with people, the robot also need to know about the humans around it: where they are and what are their characteristics.

Computer vision using standard cameras is one commonly used method to get information about people. Observing people with cameras in a public space can be in conflict with the request for keeping their privacy, since a person’s identity can easily be identified in a video. We would prefer to have robots in public which use sensors that cause less privacy concerns.

Depth sensors measure the distance from the sensor to the object (often called depth or range measurements), instead of color information like standard RGB cameras. As recognizing persons in depth measurements is arguably harder than in color images, these sensors give better protection of privacy than standard cameras. However, many of the 3D depth sensors available nowadays have a rather limited field of view; examples of such sensors include time-of-flight cameras or structured light cameras. On the other hand, our experience with social robot services in public spaces suggests that in order to have a successful interaction, for many applications it is important for the robot to perceive persons in a wide area around itself. For example, if the robot wants to understand how people use the space [1], or to approach a person when he passes by [2], it should be able to track people when they are 5 or more meters away. This exceeds the range of reliable detection and tracking that can be achieved with a typical 3D depth sensor (see e.g., [3]).

A depth sensor which does allow larger distance sensing is a LiDAR (short for “Light Detection And Ranging”). The mechanically rotating multi-layer LiDARs that are most commonly used nowadays have both a long range and a wide field of view, often covering the full 360${}^{{}^{\circ}}$ around the sensor. This makes them a very good option for mobile and social robots that are used in public spaces, since (a) with a single sensor it is possible to accurately determine the robot’s position in the space, even in wide areas such as squares; and (b) it can be used to detect and track the position of people and other objects in a wide range around the robot. That is why we have often used onboard LiDARs in our research on social robots in public environments, for example in the works [1,2] cited above.

Just knowing where people are is not always enough for good interaction. In many cases it would be helpful if the robot knew something more about the state and features of the person it needs to interact with: where are they facing, do they hold something in the hands, do they carry a suitcase or a backpack, what is their gender, etc. Up to now, usage of LiDARs for estimating people’s features has not been studied. One reason is that, unlike autonomous cars, LiDARs have not yet been widely adopted for use on robots. This in turn is due to their price, which as of now is still quite high. In recent years there has been a substantial investment in the development of new and cheaper LiDARs [4], largely driven by the research on autonomous driving. It is therefore expected that the LiDAR prices will gradually decrease and that will lead to higher adoption for mobile robots.

Another reason why LiDARs have not been used for estimation of people’s features is that the measurements that currently available rotating 3D LiDARs give are typically rather sparse. As a result of that sparsity, it is not possible to attain an accuracy comparable to state of the art results obtained using either cameras or other depth sensors. Nevertheless, feature estimation using sparse LiDARs can still be useful. For example, in many human–robot interaction applications, even lower accuracy estimates can often be enough. Even when a different sensor is used for more accurate estimation, the wide field of view of the LiDAR can make it a valuable secondary sensor that provides a less accurate estimate of people’s features all around the robot. These are the reasons why in this work we wanted to determine whether and to what extent can current LiDAR sensors be used for estimating people’s features.

In particular, we studied the estimation of people’s body angle and gender. One reason is that both can be useful to a robot interacting with humans. One more reason is that they represent two different types of features: body angle is an example of a continually changing feature, whereas gender is a static, non-changing one. We were interested in seeing how the estimation of both these types can be done using LiDAR data.

## 2. Related Work

### 2.1. 3D LiDAR Sensing Technology

As we already mentioned, the mechanically rotating multi-layer LiDAR is at this moment the most frequently used type of real-time LiDAR sensor. It produces a 3D scan of the environment by mechanically rotating multiple laser detectors. Since the position of the laser detectors is fixed, the resolution of the scan in the vertical direction (perpendicular to the direction of rotation) is determined by the number and arrangement of the detectors. The sensor models produced by the company Velodyne LiDAR have been typical representatives of this category, although more recently other companies started marketing LiDARs of this type. More details about the principle of operation of rotating multi-layer LiDAR will be described on the example of the sensor that we used in this work in Section 3.1.

Other types of scanning principles can be used for LiDARs [5], and importantly, they also have different sensing characteristics. First, apart from rotating the laser source and detectors, the direction of the laser beam can also be changed using mechanically actuated mirrors. This is often done using microelectromechanical (MEMS) actuators, which allows more integrated and low cost solutions [6]. Due to the need for space to actuate mirrors the field of view is typically limited; however, some sensor models combine rotation and actuated mirrors to achieve almost full 360${}^{{}^{\circ}}$ field of view [7].

Next, there are so called Flash LiDARs, which use lenses to emit a wide laser beam and then detect the returning light using a 2D array of detectors [8]. This is the same principle of operation as standard time-of-flight (ToF) depth cameras, but since it uses lasers instead of normal light, the Flash LiDAR is able to achieve higher measurement accuracy and larger ranges of operation. However, it generally has a field of view similar to ToF depth cameras. Optical phased arrays are yet another LiDAR operating principle that is currently being developed [9].

All the above alternative LiDAR types are still under active development, and such sensors have only recently started appearing on the market. Thus, unless noted otherwise, in this paper when talking about a 3D LiDAR we will be referring to the rotating multi-layer LiDAR.

### 2.2. Using Mobile 3D LiDARs

In mobile robotics, 3D LiDARs have most often been studied for the tasks of self localization and building of the environment map [10,11,12]. In these works LiDAR was used to build a map of the environment using a simultaneous localization and mapping (SLAM) algorithm. Once the 3D map is built, the robot can use it to to localize itself, by comparing the map with the LiDAR measurements [13].

Another task for which LiDARs have been used is to detect and recognize objects around robots. Especially in the field of autonomous driving there are many studies on the recognition of cars, pedestrians or other objects around the vehicle. For recent reviews of the recognition and classification of objects using mobile LiDAR sensors see [14,15,16]. (An often used approach is also to combine LiDARs with other sensors such as cameras [17,18,19]; however, as noted in the introduction in this work we focused on using only LiDARs.)

More closely related to our work, several researchers have used 3D LiDARs for the detection of pedestrians around the autonomous vehicles [20,21,22,23]. For autonomous driving, this is an important sensing capability as it is needed to avoid collisions with pedestrians. Just in the last two years several new datasets with 3D LiDAR data appeared [24,25,26,27] and this is going to further drive the research on the detection and classification of pedestrians and other objects in 3D LiDAR scans. Apart from only detecting people, the tracking of people with a 3D LiDAR mounted on a car or robot is also frequently studied [1,23,28,29].

### 2.3. Detecting Human Features Based on Depth Data

Depth sensors such as stereo cameras, time-of-flight cameras, structured light cameras, etc. (often referred to as range cameras or depth cameras), typically have a better resolution than a 3D LiDAR, but a smaller sensing range. The depth camera is often combined with an RGB camera, and this combined sensor is usually called RGB-D camera (an example is the Microsoft Kinect sensor [30]).

There are several studies that use either depth or RGB-D cameras for person detection [31] or tracking [3,32,33]. Moreover, in a number of works they are also used for the recognition of people’s characteristics. For example, Linder et al. studied the use of RGB-D data for recognition of gender in [34], and also of other human characteristics in [35]. A related study [36] proposed a neural network-based recognition of the mobility aids that people are using in a hospital environment.

For estimating the body orientation, one approach is to use a RGB-D camera and a human skeleton detector (e.g., [37], or the one provided by the Kinect sensor [30,38]). The body angle can then be calculated from the estimated positions of body joints. Alternatively, the body orientation can be estimated directly from the depth image, such as in [39], where a deep neural network-based approach was shown to have better accuracy than skeleton-based approaches.

3D LiDARs were used in a number of works for object classification [14], and often the task included determining whether the object is a pedestrian, a bicyclist or a car [40,41,42]. Yet, there is basically no research on recognizing features of the tracked people, which is what we aimed to do in this study.

### 2.4. Using Simulations for Learning

Using simulations to acquire a large dataset of synthetic depth data has proven to be a efficient way to obtain large datasets for training an estimator. One application where this approach is commonly used is detection of human body pose [38,43], or the pose of parts of the body, such as hands [44]. We use a similar approach to collect a large number of synthetic samples of LiDAR data.

## 3. Collecting Datasets

### 3.1. Velodyne HDL-32E LiDAR

The target platform in this work was a mobile social robot equipped with a LiDAR. The choice of the robot is not really important for the conclusions in this work (in the experiments we used the robots Robovie II and Robovie R3). However, the results do depend on the characteristics of the sensor.

**Figure 1:**
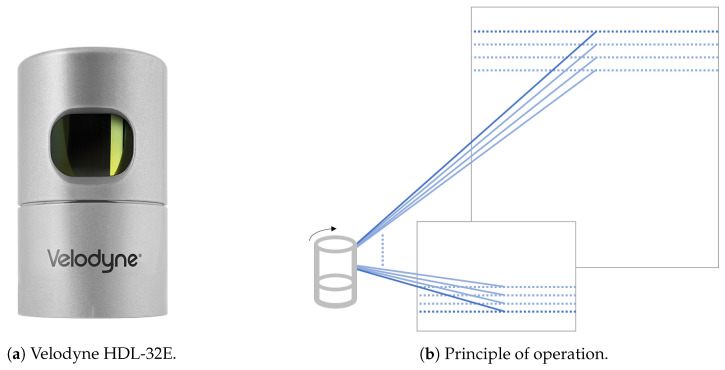
Rotating multi-layer laser scanner (Light Detection And Ranging (LiDAR)) used in this work.

In this work we used the Velodyne HDL-32E LiDAR sensor, Figure 1a. During all the data collections we used it mounted on a pole in the back of the robot, at the height of 1.35 m. The Velodyne HDL-32E [45] is a rotating multi-layer laser scanner. It scans the environment using a set of 32 laser range detectors which rotate together, as illustrated in Figure 1b. The measurement data contains 3D points which represent the surface of the scanned objects (a 3D “point cloud”).

Velodyne HDL-32E has a sensing range of up to 100 m. This large sensing range is typical for LiDARs and it is much larger then what can be achieved with other 3D depth sensors, such as time-of-flight (ToF) cameras or structured light cameras. For example, the Microsoft Kinect (structured light camera) or Microsoft Kinect v2 (ToF camera) sensors [30] can provide reliable measurements only up to around 5 m. The long range allows the use of the LiDAR sensor for accurately localizing the robot, even in wide areas like public squares. On the other hand, it also makes possible the tracking of persons while they are still relatively far from the robot, which can be important for a number of applications in human–robot interaction where the robot needs to predict the future movement of the person.

The Velodyne HDL-32E sensor scans 360${}^{{}^{\circ}}$ around itself in 32 scanning layers. In each layer it scans the area with the resolution of approximately 0.16${}^{{}^{\circ}}$ (when rotating at 10 Hz). Vertically the layers span 40${}^{{}^{\circ}}$, so the angle between layers is 1.33${}^{{}^{\circ}}$. Note how the scans are much sparser vertically than horizontally. As Figure 1b, the scans also become farther apart at larger distances.

### 3.2. Obtaining People Point Clouds from LiDAR Data

When the robot is moving in the environment, it is not trivial to determine which part of the measurement from the LiDAR sensor belongs to which person. The basic procedure that we used to extract the part belonging to each person consisted of the following steps: localize the robot with respect to a prebuilt map; determine the parts of the measurement which belong to people; and then continually track each person. We briefly describe the whole procedure here.

First, we built the map of the environment. We manually drove the robot inside the environment and collected LiDAR measurements. Than we used the SLAM 6D method from [12] to create a 3D map of the environment. Next, for localizing the robot we used a particle filter based estimator, which matched the LiDAR measurements with the 3D map [13].

Once the robot is localized, the part of the scan which was not matched with the map can be extracted. In order to get the part of the scan which belongs to a particular person we dived the scan into clusters. Since the scanner has a low resolution in the vertical direction, the difference in height between points in different scanning layers can be significant, so the clustering was done based on the Euclidean distance in the ground projection. The clusters that had too little points (less than 20) were ignored.

Each obtained cluster was represented with a single point with ground projection (x,y) coordinates equal to the mean value of all the points in the cluster, and the height z equal to the height of the highest point. For continually tracking people we used a set of particle filters, with one filter for each tracked person. The state of each particle was given by the position, speed *v* and movement direction angle θ of the person. The matching between the extracted cluster and the already tracked person (particle filter) is done based on the 2D Euclidean distance in the position, and for clusters that were not matched a new particle filter was created. The rest of the implementation is similar to [1,33], so refer to those works for details.

We used the above procedure for collecting all datasets of LiDAR data described in the following section. The only exception is the lab dataset, where the robot was not moving so in that case there was no need to build a map and run localization.

### 3.3. Datasets

For training and testing the estimator for people’s features, we used three different sets of LiDAR data. Collecting sensor measurements of people in public spaces and then manually labeling them is a laborious task. We therefore wanted to know if it is possible to efficiently train the estimator on data that can be more easily collected or obtained. We tested two alternative data sources:

- Artificially created LiDAR dataset (simulated dataset, Section 3.3.1)
- LiDAR data collected in a laboratory environment (lab dataset, Section 3.3.2)

In addition we collected LiDAR data in the real world, which we used for the testing of the estimation results (real world dataset in Section 3.3.3).

#### 3.3.1. Simulated Dataset

One dataset consisted of artificial LiDAR data that we created using a simulator. We have developed a simulator for studying the human–robot interactions in public environments [46]. The simulator is based on the MORSE robot simulator [47], which provides models of different sensors, actuators and robots. We have extended it to add the possibility to simulate many pedestrians moving through the environment. In addition, we improved the simulation of 3D LiDAR sensors like Velodyne HDL-32E to make the simulation or the measurements faster and more realistic.

We also prepared 110 different 3D models of pedestrians of both genders, including 12 models of children. These models were manually created to have a variation in clothing, hairstyle, age, gender and worn accessories. For each simulated person, one of the 3D models was chosen at random.

**Figure 2:**
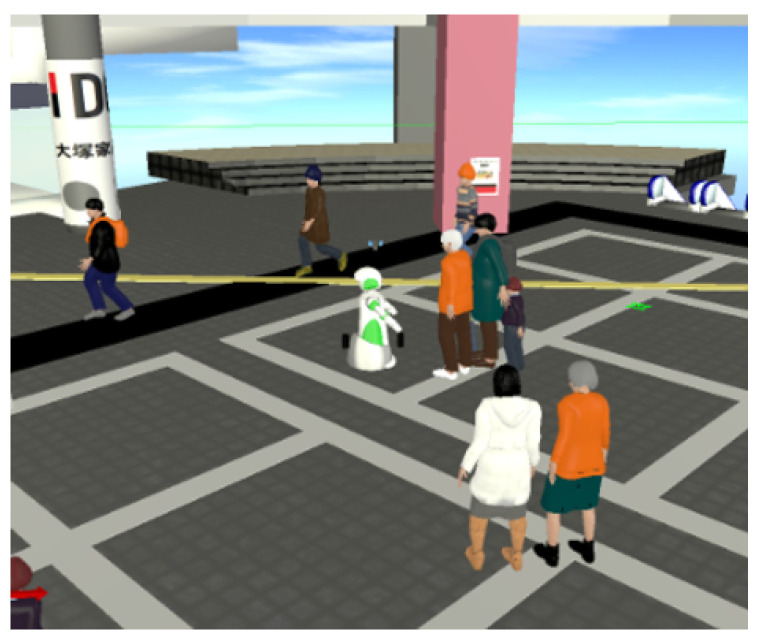
Using a simulator to collect artificial LiDAR measurements of people.

As a result, we were able to run a reasonably realistic simulation of situations that a social robot would encounter in a public environment, see Figure 2. In the simulation one robot with a LiDAR sensor was moving in the environment (a model of an actual shopping mall), and people would pass nearby and sometimes approach the robot. During the simulation we collected both sensor data for the simulated pedestrians, as well as ground-truth data like position and body orientation.

The resulting dataset contains around 1.37 million samples in total, where a sample is one LiDAR measurement of one simulated person.

#### 3.3.2. Lab Dataset

**Figure 3:**
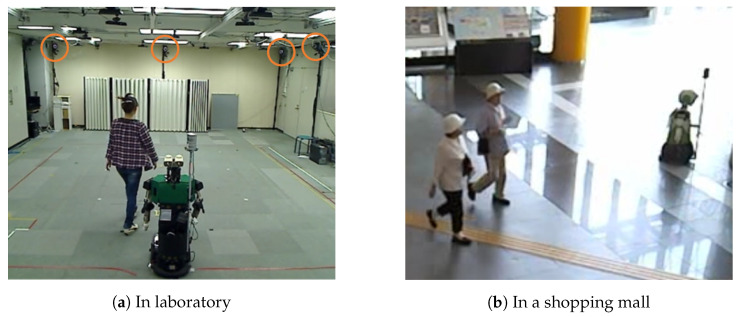
Data collection using actual LiDAR sensor.

The second dataset contains actual LiDAR data collected in a laboratory environment. We invited subjects to the lab and let them move inside a room. During that time we used a Velodyne HDL-32E sensor mounted on top of a robot to collect the range data, Figure 3a. A motion tracking system was installed in the same room (the motion tracker system cameras are circled in the photo in Figure 3a). During the data collection the subjects were wearing motion tracker markers on their shoulders and head, which allowed us to obtain also the ground truth for the subjects’ position and body angle.

We repeated the data collection with 15 subjects (6 male, 9 female). The resulting dataset contains about 75 thousand samples in total.

#### 3.3.3. Real World Dataset

Finally, we also collected data in an actual populated environment—the ATC shopping mall in Osaka in Japan, shown in Figure 3b. During the data collection, a robot with the Velodyne LiDAR was remotely controlled by an operator.

In order to get the ground truth for gender we used manual labeling. The space where we did the collection was also covered with cameras which allowed us to collect videos. We used these videos synchronized with the result of tracking to label the people.

For continually changing variables such as the body angle direct measurement and manual labeling are both difficult in the real world. Instead we assumed that the body angle is equal to the person’s walking direction (which we estimated by continually tracking each person, as described in Section 3.2). This is a reasonable assumption when humans walk fast enough (used also e.g., in [48]). Thus, for people who were walking we set the ground truth for their body angle to the estimated direction of movement. Since the tracking of people is more stable when the robot is not moving, in order to get a better estimate of the body angle, during the data collection we kept the robot mostly stationary and changed its position only from time to time. For people who were standing there was no easy way to determine their body angle so we did not include them in the dataset.

We did the collection during two days, for a total of 5 h (42 min during the first day, 220 min during the second day). For about 1500 pedestrians we obtained labels of gender as well as body angles. The resulting dataset contains about 93 thousand samples in total.

The preprocessed datasets of LiDAR data (simulated, lab and real world) and code samples for using them are available to other researchers upon request. The datasets contain the person point clouds with the corresponding gender and body angle features. Readers interested in using them can contact the corresponding author.

## 4. Estimation of Features

**Figure 4:**
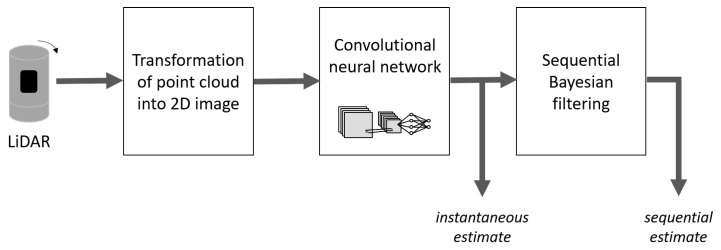
Flow of feature estimation.

Figure 4 shows an overview of the proposed method for estimating the body angle or gender from LiDAR measurements.

### 4.1. Mapping of Point Clouds to 2D Images

**Figure 5:**
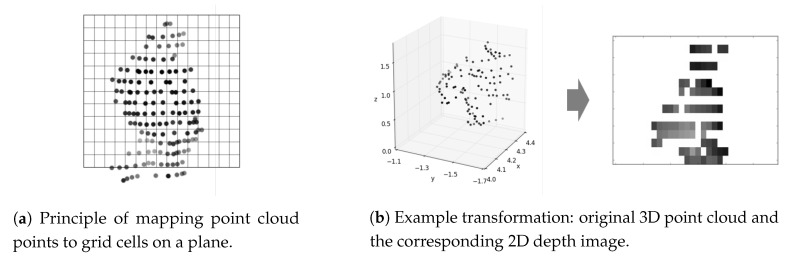
Transformation of LiDAR point cloud into a 2D depth image.

The cluster of scan points (point cloud) belonging to a person is first transformed into a 2-dimensional image. The procedure is illustrated in Figure 5a.

We start by placing the image plane perpendicular to the ground and oriented towards the sensor, i.e., the plain is perpendicular to the line connecting the sensor and the point cloud center (the average position of all points in the point cloud). The plane also passes through the point cloud center. The width and the height of the plane are kept fixed for all people. The plane is horizontally centered at the point cloud center, and its top is set to be slightly above the highest point in the point cloud.

The thus created image plane is divided into a homogeneous grid of pixels. The points in the point cloud are projected onto the image plane and for each pixel a value between 0 and 1 is assigned. For pixels into which no point fell the value is set to 0. For the rest of the pixels the value is calculated based on the average distance to the plane of all the points that fall into that pixel. The distances are defined as positive if the points are closer to the sensor then the image plane, or negative otherwise. The average distance of 0 is mapped to the value of 0.5, and the maximum positive and negative distances to 1 and 0, respectively, where the maximum distances are set to ±0.5 m.

The result of such mapping is a gray-scale image, as shown in Figure 5b.

We tested with different sizes and resolutions for the 2D image plane. The size that worked well was 1 meter horizontally by 0.6 m vertically, which included the person’s head and upper torso. For the resolution we settled on using 30 pixels horizontally by 15 pixels vertically. While lower resolutions diminished the estimation accuracy, higher resolutions did not result in significant improvements.

### 4.2. Convolutional Neural Networks for Estimation

After the above mapping, the problem becomes equivalent to estimating features from a 2D gray-scale image. For the estimation we used a standard 2D Convolutional Neural Network (CNN).

We used separate CNNs for body angle and gender estimation. We ran tests using different structures and layers sizes of the CNN and finally settled on a simple network structure with two convolutional layers followed by two fully connected layers, as using more layers did not result in significant improvements in the performance. For both features, in the convolutional layers we used standard 2D convolution operations followed by rectified linear unit (ReLU) activation functions, and the first fully connected layer also had a ReLU activation function. After each layer we used batch normalization, as well as dropout to prevent overfitting.

The numbers of neurons in each layer were determined using random search. For body angle estimation we had 64 (first convolutional layer), 96 (second convolutional layer) and 96 in the first fully connected layer. As angles have a discontinuity at ±π, instead of letting the neural network estimate the body angle values directly, we estimated the sine and cosine of the body angle. Thus the last layer had 2 neurons, both with a linear activation function. The resulting angle was calculated by taking the arctangent of the ratio of the estimated sine and cosine values.

For the gender estimations the determined optimal numbers of neurons was 32, 64 (convolutional part) and 128 (first fully connected layer). Since this is a binary estimation problem, the output layer contained two fully connected neurons with a softmax activation function. The output was therefore a probability distribution over the two possible gender values: male or female.

For all datasets and both CNNs, the training was done using the same procedure. We used the Adam optimizer, with mean squared error as loss function for estimating the body angle, and categorical crossentropy for the gender. The batch size was set to 512. The maximum number of training epochs was set to 200, and we used 10% of the training data for validation and used early stopping to stop the training when there was no improvement of the validation loss for 10 epochs. (Full details about these techniques can be found in references such as [49].) The implementations were made with Tensorflow using the Keras API.

We should note that it is also possible to use 3D point clouds from the LiDAR directly, i.e., without transforming them to 2D depth images, for example by using 3D convolutional neural networks. This includes first transforming the point cloud into a voxel grid (a 3D occupancy grid) of fixed size and using it as input to a 3D convolution. Since the LiDAR scan points are only on the surface of the person, and only on one side, most of the voxels end up being empty, and as a consequence the input data is quite sparse. In our tests this made the training of 3D CNNs less stable (having more variation in the training curves), compared to 2D CNNs. As there was no significant difference in the achieved accuracy, we opted for using 2D CNNs.

### 4.3. Sequential Bayesian Estimation

The convolutional neural networks give us an estimate of the body angle or gender for each obtained LiDAR point cloud. That is, for each person in the LiDAR scan we obtain values for the body angle and gender estimates. These estimates are based on a single measurement, so we refer to them as instantaneous estimates.

However, as explained in Section 3.2, we are also continuously tracking each person between measurements. It is therefore possible to combine the instantaneous estimates with the previous estimates for the same person. To achieve that we used sequential Bayesian estimation (Bayes filters) [13]. We will call the result of applying sequential Bayesian estimation the sequential estimate.

Body angle estimates take continuous values. Under the assumption that the noise in the estimate from the CNN is Gaussian, the Bayes filter turns into the so called Kalman filter [13]. In the Kalman filter the estimate is described with two values: the state *x* and the covariance *P*, which represents the uncertainty about the estimate. Since, in our case, body angle was a scalar, the state and covariance values were also scalars.

Using a simple process model which states that the angle stays the same between two LiDAR measurements, the prediction step of the Kalman filter becomes:

(1) $$\begin{array}{cc} x_{k}^{\prime } & =x_{k-1}, \end{array}$$

(2) $$\begin{array}{cc} P_{k}^{\prime } & =P_{k-1}+Q, \end{array}$$

where Q represented the covariance of the process noise (assumed constant).

In the update step, the body angle estimate from the CNN (denoted $z_{k}$) is used to obtain the recursive estimate at step *k* using the following calculation:

(3) $$\begin{array}{cc} K_{k} & =P_{k}^{\prime }/\left(P_{k}^{\prime }+R\right),\;\left(“\mathrm{Kalman}\;\mathrm{gain}”\right) \end{array}$$

(4) $$\begin{array}{cc} y_{k} & ={\left(z_{k}-x_{k}^{\prime }\right)}_{\in (-\pi ,\pi ]},\;\left(“\mathrm{residual}”\right) \end{array}$$

(5) $$\begin{array}{cc} x_{k} & ={\left(K_{k}\cdot y_{k}\right)}_{\in (-\pi ,\pi ]}, \end{array}$$

(6) $$\begin{array}{cc} P_{k} & =\left(1-K_{k}\right)P_{k}^{\prime }. \end{array}$$

The value of R represents the covariance of the measurement noise (also assumed constant).

Note that the standard Kalman filter assumes that the prediction and measurement models are linear, and strictly speaking, this is not true for angles since they have a discontinuity at ±π. However, this discontinuity can be avoided computationally by transforming the angles after calculations to the interval (−π,π]. This is used in Equations (4) and (5), where ${\left(\cdot \right)}_{\in (-\pi ,\pi ]}$ denotes the transformation of the term inside the brackets to the interval (−π,π].

For sequential estimation of binary variables such as gender, Bayesian filtering gives us a different set of predict and update steps. In the following equations $p_{k}\left(m\right)$ represents the probability of a person being male at time step *k* (the probability of being female is $p_{k}\left(f\right)=1-p_{k}\left(m\right)$). The output of CNN, the instantaneous estimate of the probability of being male is noted as $z_{k}\left(m\right)$.

For the predict step we have the following equation:

(7) $$\begin{array}{cc} p_{k}^{\prime }\left(m\right) & =\left(1-\alpha \right)p_{k-1}\left(m\right)+\alpha \cdot 0.5. \end{array}$$

This models the partial resetting of the estimated probability towards the value 0.5 (probability for both male and female is 0.5, i.e., gender is fully unknown). The value of the factor α models the uncertainty in the instantaneous estimates up to time *k*, with larger values indicating more uncertainty in old values and more trust in new values.

The update step applies the Bayes’ theorem, with $p_{k}^{\prime }\left(m\right)$ used as prior:

(8) $$\begin{array}{c} p_{k}\left(m\right)=\frac{p_{k}^{\prime }\left(m\right)z_{k}\left(m\right)}{p_{k}^{\prime }\left(m\right)z_{k}\left(m\right)+\left(1-p_{k}^{\prime }\left(m\right)\right)\left(1-z_{k}\left(m\right)\right)}. \end{array}$$

This gives us the sequential estimate for the gender.

## 5. Results

### 5.1. Evaluation Procedure

The evaluation consisted in training the CNN on one dataset and testing it on another dataset. Since we were interested in the estimation performance on actual LiDAR data, for testing we only used the lab and real world datasets. When the lab dataset was used for training we did not test on the lab dataset. In addition, we also used the combined simulated and laboratory datasets for training.

During training we used 10% of the dataset for validation and the rest for training. The validation dataset was chosen randomly, but taking care that there is no information leakage between the parts used for training and validation (for example, we trained and validated on different people, since not doing so could affect the result, in particular for gender estimation). During training we continually checked the accuracy on the validation data, and when it stopped improving we stopped the training to prevent overfitting. The same procedure was used for all evaluations.

After obtaining the instantaneous estimates, we ran the Bayes filters for each tracked person to get the sequential estimates. The appropriate parameters for the filters were determined using a grid search. For the body angle Kalman filter we used Q=0.04 and R=1. In the binary Bayesian filter for the gender we used α=0.01.

In addition, for comparison we also tried training directly on the real world actual LiDAR data. For that we trained the CNN on data from the second day of data collection in the shopping mall (see Section 3.3.3), which contained about 83% of the total data. The testing was done on the remaining 17% of data from the first collection day. It should be pointed out that these results are not directly comparable to the other results, since the testing was not done on the full dataset but only on a subset of it. To highlight that we marked them in italic in the results below.

### 5.2. Estimation of the Body Angle

The mean absolute error (MAE) of the estimated body angles are shown in Table 1. Rows correspond to the datasets used for training, whereas columns represent the different testing dataset (either lab or real world) and type of estimate (instantaneous or sequential). The last row shows the result of doing both training and testing directly subsets of the real world data, as explained above.

(Note that the body angle from the real world dataset that we compared with in these results is not an accurate value, but it is itself also an estimate, approximately calculated as the movement angle of people while walking. Hence a more appropriate name for the reported values in the table for the real world dataset would be mean absolute difference. However, for simplicity we used the term mean absolute error for all results.)

Training on the simulated and lab datasets gave relatively good accuracy. The superiority of simulated dataset, even though contains artificially created data, is probably due to its much larger size. The best result, marked bold in the table, was obtained when we trained on the combination of the artificial-but-large simulated dataset and the small-but-real lab dataset. Moreover, the obtained error seems to be even smaller than what training directly on the real world data gives. Again, we believe this is due to the large sample size of the simulated dataset.

Sequential estimates gave in general lower mean absolute errors then instantaneous ones, proving the advantage of using the Kalman filter.

**Figure 6:**
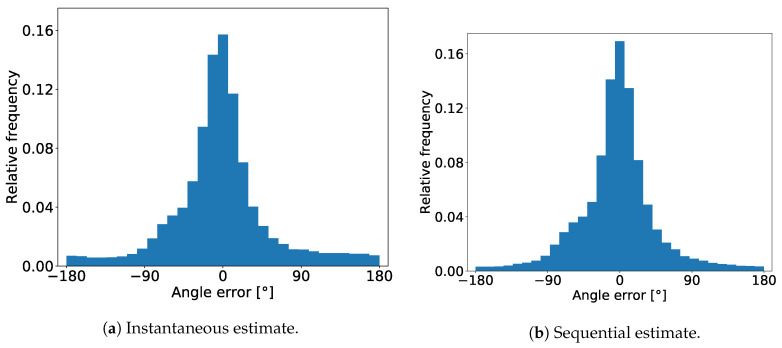
Distribution of body angle mean absolute errors.

In Figure 6 we show the distribution of the MAE on the real world dataset for the case when the simulated and lab dataset together were used for training.

The distributions for both instantaneous and sequential estimates show that the errors are concentrated around 0${}^{{}^{\circ}}$, with the majority results falling between −90${}^{{}^{\circ}}$ and +90${}^{{}^{\circ}}$. One thing that can be noticed is that there are no peaks around ±180${}^{{}^{\circ}}$, which means that the CNN estimator basically never mistakes the person’s back from the front.

Comparing the two distributions we can see that the effect of using the sequential estimates is that that the error becomes more concentrated around zero and the distribution tails become thinner.

**Figure 7:**
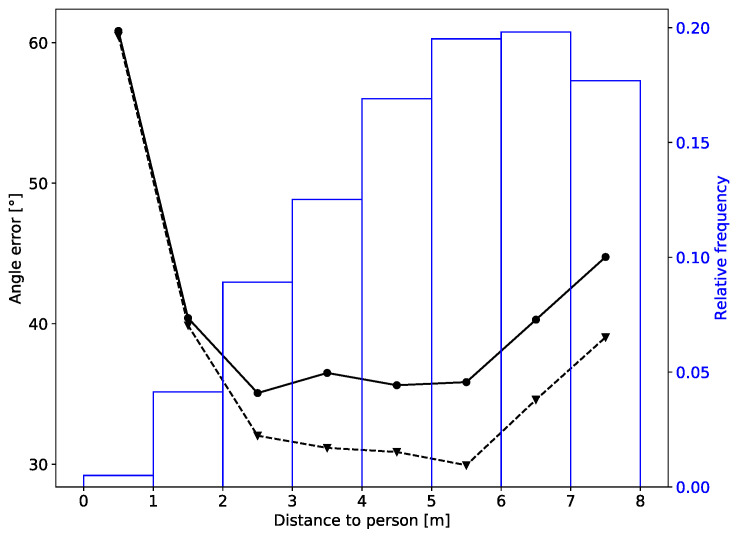
Dependence of the body angle error on the distance to the person: solid line—instantaneous estimate; dashed line—sequential estimate. The figure also shows the distribution of distances in the testing dataset (real world dataset).

Finally, we wanted to know for which distances between the LiDAR sensor and person we may expect to get more accurate estimates of the body angle. Figure 7 shows the dependence of the mean absolute error of the body angle on the distance of the person.

We can see that the best accuracy was obtained for distances between 2 and 6 m. For distances larger then 6 m the estimation error gradually increases, the most probable cause being that the LiDAR measurements become sparser. The accuracy is lower also when the person is closer that 2 m from the LiDAR. The reason for that is that the vertical field of view of the Velodyne HDL-32E sensor is only 40${}^{{}^{\circ}}$, so when a person stands close to it only part of her head and torso is scanned. This results in a different shape at the input of CNN and subsequently in a poorer estimate of the body angle. For distances below 1 m the error seems to be very large, but there are only a few samples as people very rarely came this close, so we cannot be confident in this result.

We also evaluated the average execution times of body angle estimation. They were evaluated on a laptop PC with i7-8550U CPU, 16 GB of RAM and an NVIDIA GeForce MX150 GPU, as this is a typical setup for the PC that we use for processing the Velodyne data on the robot. The calculation time for a single person was 13.4 ms when the GPU was used, and 17.2 ms when using only the CPU. Since the Velodyne sensor gives a new measurement every 100 ms, this leaves enough time for other data processing tasks (such as robot localization and people tracking).

### 5.3. Estimation of Gender

Table 2 shows the obtained gender estimation results.

For gender training on the simulated dataset did not result in a good accuracy. The likely issue is that, even though the total number of samples is large, they were all obtained from a limited number (110) of simulated 3D human models. The shape variations between those models are not nearly enough to cover the possible variations in real people, so training on them does not give a good accuracy when tested on real people. We achieved the best result when training on the lab dataset. Even though this dataset is relatively small and taken on a small number of people, it still outperformed the simulated dataset. Yet, even this accuracy of 0.68 is relatively poor and cannot be compared to the result obtained when training on real world data. Perhaps unsurprisingly, this seems to suggest that for features such as gender it is more important to have examples from many different real people than to have many samples in the dataset. The sequential estimates are again mostly better than instantaneous ones.

**Figure 8:**
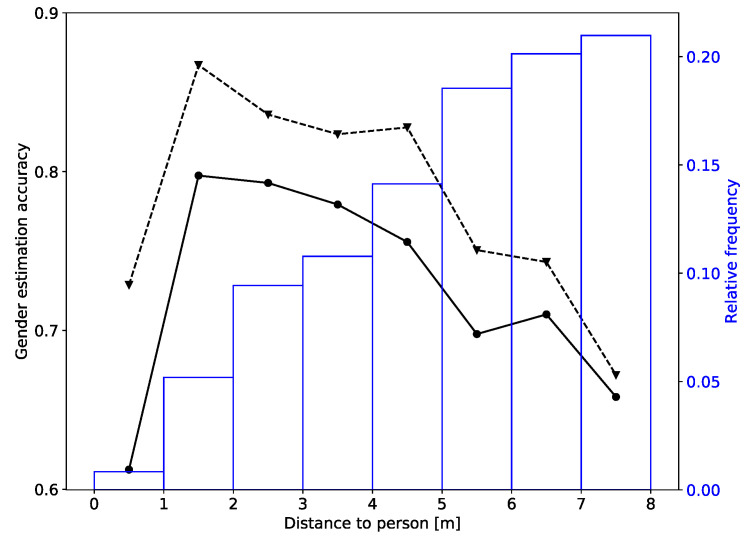
Dependence of the gender estimation accuracy on the distance to the person: solid line—instantaneous estimate; dashed line—sequential estimate. The figure also shows the distribution of the distances in the testing dataset (first day from real world dataset).

We also show the dependence of the accuracy on the distance of the person, Figure 8. Shown is the curve for the training on one collection day of the real world data and testing on the other day (last row in Table 2). We can see that the best accuracy is achieved for distances between 1 and 2 m (sequential estimate is 0.86). The accuracy stays relatively high (above 0.8) between 2 and 5 m, and than quickly falls off at larger distances. Since most samples are from people who were more than 5 m away, the total accuracy listed in the table is not so high; but if we just look at the closer distances the gender estimation accuracy is much better.

**Figure 9:**
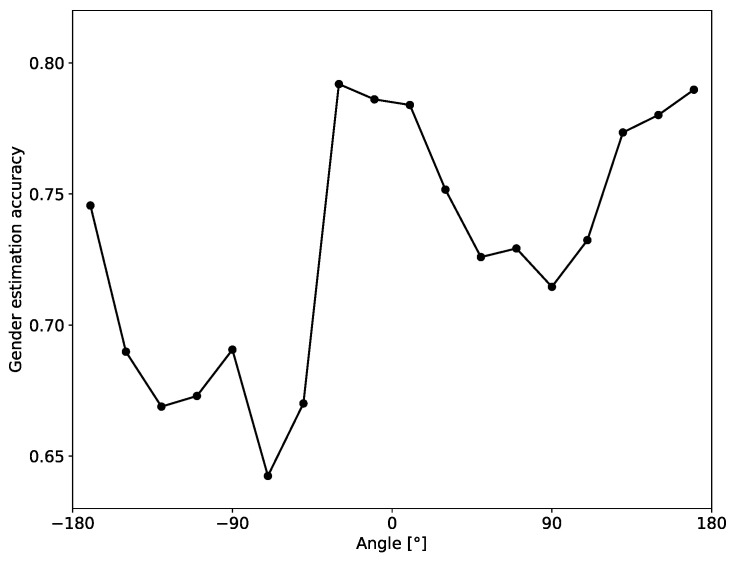
Dependence of the gender estimation accuracy (instantaneous estimate) on the body angle of the person.

In Figure 9 we also show how the accuracy of gender estimation depends on the body angle of the person. The obtained curve suggests that it is easier to recognize the gender when the person is either facing towards or away from the sensors (body angle around 0${}^{{}^{\circ}}$ and ±180${}^{{}^{\circ}}$), than when the person is detected sideways (body angle around ±90${}^{{}^{\circ}}$).

The average execution times of the CNN for gender estimation, evaluated on the same laptop PC as we used for body angle estimation, were 13.1 ms with GPU and 16.3 ms without GPU.

## 6. Discussion

### 6.1. Velodyne HDL-32E for Estimating Features

As noted in the introduction, due to the sparsity of measurements, with rotating multi-layer LiDARs we did not expect to achieve a performance comparable to using other sensors. To put the results in perspective, we compare the obtained results with the current best approaches that use other sensors.

For body orientation estimation one of the best results is presented in [39], with MAE of 6.14${}^{{}^{\circ}}$ using only depth information form a Microsoft Kinect v2 sensor. This result was obtained for ranges approximately between 1 and 6 m. (They also report a 14.69${}^{{}^{\circ}}$ error for body angle estimation based on 3D skeleton tracking from [37].) Compared to these results, the lowest error of body angle estimation using a Velodyne HDL-32E of 33.53${}^{{}^{\circ}}$ is quite high. The obvious reason for this is the difference in the resolution: Kinect has a resolution of 512 (horizontally) × 424 (vertically) pixels, with a field of view of 70.6${}^{{}^{\circ}}$× 60${}^{{}^{\circ}}$, and this gives a much denser point cloud then Velodyne, which vertically has only 32 scans in 40${}^{{}^{\circ}}$.

Although not highly accurate, the achieved accuracy of body angle estimation could still be suitable for some human-robot interaction applications. For example, it is enough for the robot can judge whether a standing person is facing away or towards it and thus decide if it should wave, approach or just ignore him. Or, it could use the angle information to plan an appropriate path to approach a person. Being able to do this for 360${}^{{}^{\circ}}$ around the robot, and with the same sensor that is used also for robot localization and tracking of people is very convenient. In cases when better body angle accuracy is needed, it will be necessary to combine Velodyne with another sensor, e.g., a depth camera.

For the estimation of gender, [34] reported the gender classification accuracy of 0.9107 using depth data from the Kinect v2 sensor (for distances approximately between 1 and 4 m from the sensor). Even though the result with Velodyne is less accurate, for people close to the robot it makes a mistake in less the 1 in 5 people, so it could still be useful for interactive services (as noted in [50], a small number of robot mistakes is usually not judged harshly by interacting humans). However, if one needs to have better accuracy the estimate may need to be combined with other sensors, such as an RGB camera or sound based gender recognition.

### 6.2. Training on Artificial Data

Using artificial LiDAR data from simulated human models turned out to be a good way to improve the accuracy of body angle estimation. It seems that the ability to gather a large number of samples that cover well all the whole range of possible values is important for estimating continuous features like the body angle, even if the simulated models are not completely realistic. We expect that the same would be true if we wanted to estimate other continuously changing features, such as pose of arm or leg, or head angle.

On the other hand, for gender it seems that having many realistic examples was more important then just the number of samples. We imagine that this would be similar for other static features, such as haircut or clothes.

### 6.3. Generalizability to Different LiDARs

Although in this work we used one specific LiDAR model, the same methodology for training estimators should be applicable to other rotating 3D LiDAR models as well as other types of 3D LiDARs. For sensors with similar characteristics it may also be possible to use directly the estimators trained on Velodyne HDL-32E; however, this would need to be confirmed. As shown in the results, the accuracy of the estimation depends on the sparsity and diminishes as measurements become sparser at larger distances. We thus expect that LiDARs with higher resolution of measurements would achieve even better accuracy.

## Figures and Tables

**Table 1:** Mean absolute error of the body angle estimate for different training and testing datasets.

	Testing Data $\overset{}{\to }$	Lab		Real World	
		Instantaneous	Sequential	Instantaneous	Sequential
Training data	Simulated	26.01${}^{{}^{\circ}}$	26.78${}^{{}^{\circ}}$	40.88${}^{{}^{\circ}}$	35.46${}^{{}^{\circ}}$
	Lab	-	-	47.35${}^{{}^{\circ}}$	39.23${}^{{}^{\circ}}$
	Simulated + Lab	-	-	**38.59${}^{{}^{\circ}}$**	**33.53${}^{{}^{\circ}}$**
	*Real World*	-	-	*41.41* ${}^{{}^{\circ}}$	*41.18* ${}^{{}^{\circ}}$

**Table 2:** Accuracy of gender classification for different training and testing datasets.

	Testing Data $\overset{}{\to }$	Lab		Real World	
		Instantaneous	Sequential	Instantaneous	Sequential
Training data	Simulated	0.7543	0.7407	0.5383	0.5559
	Lab			**0.6559**	**0.6817**
	Simulated + Lab	-	-	0.6099	0.6513
	*Real World*	-	-	*0.7224*	*0.7653*

## References

[1] Karunarathne D., Morales Y., Kanda T., Ishiguro H. (2020). Understanding a public environment via continuous robot observations. Robot. Auton. Syst..

[2] Mizumaru K., Satake S., Kanda T., Ono T. Stop doing it! Approaching strategy for a robot to admonish pedestrians. Proceedings of the 2019 14th ACM/IEEE International Conference on Human-Robot Interaction (HRI).

[3] Munaro M., Menegatti E. (2014). Fast RGB-D people tracking for service robots. Auton. Robot..

[4] Liu K., Li Y. (2020). New patent maps to visualize worldwide patent strength of competitors on LiDAR system. J. Multidiscip. Eng. Sci. Stud..

[5] Li Y., Ibanez-Guzman J. (2020). Lidar for autonomous driving: The principles, challenges, and trends for automotive lidar and perception systems. arXiv.

[6] Yoo H.W., Druml N., Brunner D., Schwarzl C., Thurner T., Hennecke M., Schitter G. (2018). MEMS-based lidar for autonomous driving. e & i Elektrotechnik Und Informationstechnik.

[7] Hokuyo YVT-35LX 3D Scanning Range Finder. https://www.hokuyo-aut.jp/search/single.php?serial=224.

[8] Amzajerdian F., Roback V.E., Bulyshev A., Brewster P.F., Hines G.D. Imaging flash lidar for autonomous safe landing and spacecraft proximity operation. Proceedings of the AIAA Space and Astronautics Forum and Exposition (SPACE 2016).

[9] Eldada L., Yu T., Pacala A. (2018). Optical Phased Array Lidar System and Method of Using Same. US Patent.

[10] Zhang J., Singh S. LOAM: Lidar odometry and mapping in real-time. Proceedings of the Robotics: Science and Systems Conference (RSS).

[11] Moosmann F., Stiller C. Velodyne slam. Proceedings of the 2011 IEEE Intelligent Vehicles Symposium (IV).

[12] Nüchter A., Lingemann K., Hertzberg J., Surmann H. (2007). 6D SLAM—3D mapping outdoor environments. J. Field Robot..

[13] Thrun S., Burgard W., Fox D. (2000). Probabilistic Robotics.

[14] Che E., Jung J., Olsen M.J. (2019). Object recognition, segmentation, and classification of mobile laser scanning point clouds: A state of the art review. Sensors.

[15] Arnold E., Al-Jarrah O.Y., Dianati M., Fallah S., Oxtoby D., Mouzakitis A. (2019). A survey on 3d object detection methods for autonomous driving applications. IEEE Trans. Intell. Transp. Syst..

[16] Li Y., Ma L., Zhong Z., Liu F., Cao D., Li J., Chapman M.A. (2020). Deep Learning for LiDAR Point Clouds in Autonomous Driving: A Review. arXiv.

[17] Chen X., Ma H., Wan J., Li B., Xia T. Multi-view 3d object detection network for autonomous driving. Proceedings of the IEEE Conference on Computer Vision and Pattern Recognition (CVPR).

[18] Oh S.I., Kang H.B. (2017). Object detection and classification by decision-level fusion for intelligent vehicle systems. Sensors.

[19] Wang Z., Zhan W., Tomizuka M. Fusing bird’s eye view lidar point cloud and front view camera image for 3d object detection. Proceedings of the 2018 IEEE Intelligent Vehicles Symposium (IV).

[20] Kidono K., Miyasaka T., Watanabe A., Naito T., Miura J. Pedestrian recognition using high-definition LIDAR. Proceedings of the 2011 IEEE Intelligent Vehicles Symposium (IV).

[21] Li K., Wang X., Xu Y., Wang J. (2015). Density enhancement-based long-range pedestrian detection using 3-D range data. IEEE Trans. Intell. Transp. Syst..

[22] Navarro P.J., Fernandez C., Borraz R., Alonso D. (2017). A machine learning approach to pedestrian detection for autonomous vehicles using high-definition 3D range data. Sensors.

[23] Yan Z., Duckett T., Bellotto N. (2020). Online learning for 3D LiDAR-based human detection: Experimental analysis of point cloud clustering and classification methods. Auton. Robot..

[24] Patil A., Malla S., Gang H., Chen Y.T. The H3D dataset for full-surround 3d multi-object detection and tracking in crowded urban scenes. Proceedings of the 2019 IEEE International Conference on Robotics and Automation (ICRA).

[25] Chang M.F., Lambert J., Sangkloy P., Singh J., Bak S., Hartnett A., Wang D., Carr P., Lucey S., Ramanan D. Argoverse: 3D tracking and forecasting with rich maps. Proceedings of the IEEE Conference on Computer Vision and Pattern Recognition (CVPR).

[26] Sun P., Kretzschmar H., Dotiwalla X., Chouard A., Patnaik V., Tsui P., Guo J., Zhou Y., Chai Y., Caine B. Scalability in perception for autonomous driving: Waymo open dataset. Proceedings of the IEEE/CVF Conference on Computer Vision and Pattern Recognition (CVPR).

[27] Carballo A., Lambert J., Monrroy A., Wong D., Narksri P., Kitsukawa Y., Takeuchi E., Kato S., Takeda K. (2020). LIBRE: The multiple 3d lidar dataset. arXiv.

[28] Spinello L., Luber M., Arras K.O. Tracking people in 3D using a bottom-up top-down detector. Proceedings of the 2011 IEEE International Conference on Robotics and Automation (ICRA).

[29] Häselich M., Jöbgen B., Wojke N., Hedrich J., Paulus D. Confidence-based pedestrian tracking in unstructured environments using 3D laser distance measurements. Proceedings of the 2014 IEEE/RSJ International Conference on Intelligent Robots and Systems.

[30] Kinect for Windows. https://developer.microsoft.com/en-us/windows/kinect/.

[31] Spinello L., Arras K.O. People detection in RGB-D data. Proceedings of the 2011 IEEE/RSJ International Conference on Intelligent Robots and Systems.

[32] Luber M., Spinello L., Arras K.O. People tracking in RGB-D data with on-line boosted target models. Proceedings of the 2011 IEEE/RSJ International Conference on Intelligent Robots and Systems.

[33] Brščić D., Kanda T., Ikeda T., Miyashita T. (2013). Person tracking in large public spaces using 3-D range sensors. IEEE Trans. Hum. Mach. Syst..

[34] Linder T., Wehner S., Arras K.O. Real-time full-body human gender recognition in (RGB)-D data. Proceedings of the 2015 IEEE International Conference on Robotics and Automation (ICRA).

[35] Linder T., Arras K.O. Real-time full-body human attribute classification in RGB-D using a tessellation boosting approach. Proceedings of the 2015 IEEE/RSJ International Conference on Intelligent Robots and Systems (IROS).

[36] Kollmitz M., Eitel A., Vasquez A., Burgard W. (2019). Deep 3D perception of people and their mobility aids. Robot. Auton. Syst..

[37] Zimmermann C., Welschehold T., Dornhege C., Burgard W., Brox T. 3d human pose estimation in rgbd images for robotic task learning. Proceedings of the 2018 IEEE International Conference on Robotics and Automation (ICRA).

[38] Shotton J., Fitzgibbon A., Cook M., Sharp T., Finocchio M., Moore R., Kipman A., Blake A. Real-time human pose recognition in parts from single depth images. Proceedings of the IEEE Conference on Computer Vision and Pattern Recognition (CVPR).

[39] Lewandowski B., Seichter D., Wengefeld T., Pfennig L., Drumm H., Gross H.M. Deep orientation: Fast and robust upper body orientation estimation for mobile robotic applications. Proceedings of the 2019 IEEE/RSJ International Conference on Intelligent Robots and Systems (IROS).

[40] Teichman A., Levinson J., Thrun S. Towards 3D object recognition via classification of arbitrary object tracks. Proceedings of the 2011 IEEE International Conference on Robotics and Automation (ICRA).

[41] Wang D.Z., Posner I., Newman P. What could move?. Finding cars, pedestrians and bicyclists in 3D laser data. In Proceedings of the 2012 IEEE International Conference on Robotics and Automation (ICRA).

[42] Zhao J., Xu H., Liu H., Wu J., Zheng Y., Wu D. (2019). Detection and tracking of pedestrians and vehicles using roadside LiDAR sensors. Transp. Res. Part Emerg. Technol..

[43] Varol G., Romero J., Martin X., Mahmood N., Black M.J., Laptev I., Schmid C. Learning from synthetic humans. Proceedings of the IEEE Conference on Computer Vision and Pattern Recognition (CVPR).

[44] Malik J., Elhayek A., Nunnari F., Varanasi K., Tamaddon K., Heloir A., Stricker D. Deephps: End-to-end estimation of 3d hand pose and shape by learning from synthetic depth. Proceedings of the 2018 International Conference on 3D Vision (3DV).

[45] Velodyne HDL-32E. https://velodynelidar.com/products/hdl-32e/.

[46] Satake S., Kaczmarek T., Brščić D., Kanda T. Facilitating software development for mobile social robots by simulating interactions between a robot and pedestrians. Proceedings of the 2019 14th ACM/IEEE International Conference on Human-Robot Interaction (HRI).

[47] The MORSE Simulator. https://www.openrobots.org/morse/.

[48] Willems J., Corbetta A., Menkovski V., Toschi F. (2020). Pedestrian orientation dynamics from high-fidelity measurements. arXiv.

[49] Goodfellow I., Bengio Y., Courville A. (2016). Deep Learning.

[50] Mirnig N., Stollnberger G., Miksch M., Stadler S., Giuliani M., Tscheligi M. (2017). To err is robot: How humans assess and act toward an erroneous social robot. Front. Robot. AI.

